# Quantitative analysis of human endogenous retrovirus-K transcripts in postmortem premotor cortex fails to confirm elevated expression of HERV-K RNA in amyotrophic lateral sclerosis

**DOI:** 10.1186/s40478-019-0698-2

**Published:** 2019-03-18

**Authors:** Jeremy A. Garson, Louise Usher, Ammar Al-Chalabi, Jim Huggett, Edmund F. Day, Adele L. McCormick

**Affiliations:** 1 Division of Infection and Immunity, University College London, London, UK; 2 National Transfusion Microbiology Laboratories, NHS Blood and Transplant, Colindale, London, UK; 3 School of Life Sciences, University of Westminster, London, UK; 4 Maurice Wohl Clinical Neuroscience Institute, King’s College London, London, UK; 5 Molecular and Cell Biology Team, LGC, Teddington, UK; 6 School of Biosciences and Medicine, Faculty of Health and Medical Science, University of Surrey, Guildford, UK

**Keywords:** Amyotrophic lateral sclerosis, ALS, Human endogenous retrovirus, HERV-K, HERV-W, RNA, Premotor cortex

## Abstract

Over the past two decades a number of studies have demonstrated activity of the retroviral enzyme reverse transcriptase in the serum of patients with sporadic amyotrophic lateral sclerosis (ALS). Known human *exogenous* retroviruses such as HIV-1 have been eliminated as possible sources of this activity and investigators have therefore considered the possibility that human *endogenous* retroviruses (HERVs) might be involved. HERV-K (HML-2) is the most recent retroviral candidate to be proposed following the observation of elevated HERV-K expression in cortical and spinal neurons of ALS patients and the demonstration of HERV-K envelope protein neurotoxicity in vitro and in transgenic mice. This retroviral hypothesis is an attractive one, not least because it raises the possibility that ALS might become treatable using antiretroviral drugs. In the present study we have attempted independent confirmation of the observation that HERV-K RNA levels are elevated in ALS brain. Total RNA was extracted from the postmortem premotor cortex of 34 patients with ALS and 23 controls. Quantitative real-time reverse transcription PCR (RT-qPCR) was performed according to the MIQE guidelines using HERV-K *gag, pol* and *env* primer sets. Data was analysed by the 2^-∆∆Ct^ method with normalisation against two reference genes, *GAPDH* and *XPNPEP1*. Geometric mean HERV-K RNA expression levels in the premotor cortex of ALS patients were not found to be different from the expression levels in non-ALS controls. Our findings do not confirm the recently reported association between elevated cortical HERV-K RNA levels and ALS, and thus raise doubts about the role of this endogenous retrovirus in ALS pathogenesis. The results of this study may have implications for ongoing clinical trials aiming to suppress HERV-K activity with antiretroviral drugs.

**Electronic supplementary material:**

The online version of this article (10.1186/s40478-019-0698-2) contains supplementary material, which is available to authorized users.

## Introduction

Amyotrophic lateral sclerosis (ALS), also known as motor neuron disease, is a fatal neurodegenerative disease characterised by loss of motor neurons from the brain and spinal cord. In Europe and the USA the incidence is about 2 cases per 100,000 person-years and survival post diagnosis is typically 3 to 5 years. In about 5–10% a family history of ALS may be obtained in a first or second degree relative. Variants causative of or associated with ALS have been identified in at least 25 different genes, and in people with or without a family history, but the aetiology of most apparently sporadic cases remains uncertain [6].

Numerous non-genetic environmental risk factors for apparently sporadic ALS have been proposed including electromagnetic fields, heavy metals, pesticides, smoking, dietary factors, physical trauma and viral infection [16]. Enteroviruses and herpesviruses have been considered [5, 8] but more recent attention has focused on retroviruses. Retroviruses are known to cause motor neuron disease in mice [12], and the human retroviruses HIV-1 and HTLV-1 are both capable of causing ALS-like syndromes [23, 32], which in some cases have been shown to respond well to antiretroviral therapy [1, 22].

In a series of studies looking directly for evidence of retroviral involvement in ALS we have been able to exclude known exogenous retroviruses whilst repeatedly demonstrating an increased prevalence and raised levels of reverse transcriptase activity (a generic retroviral marker) in the serum of patients with ALS [3, 4, 25, 29]. The increased prevalence of reverse transcriptase activity in ALS was subsequently confirmed independently by another group [21]. In one of our studies [29] reverse transcriptase activity was detected more frequently in the serum of unaffected blood relatives of ALS patients than in unrelated controls and spouses. This raised the possibility that the observed reverse transcriptase activity might be associated with an inherited endogenous retrovirus.

Human endogenous retroviruses (HERVs), which constitute around 8% of the human genome, are thought to be the relics of retroviral germline infections that occurred millions of years ago [14]. Most HERVs are considered inactive due to the accumulation of mutations and deletions but there is increasing evidence that some of them may be capable of expressing full-length RNA transcripts, proteins and even retroviral particles. The most recently integrated HERVs such as HERV-K (HML-2) are thought to be the most intact and potentially biologically active [30] and it was therefore of great interest when elevated HERV-K RNA levels were reported in the cerebral cortex of patients with ALS [9, 19]. In addition to increased cortical HERV-K RNA, Li et al. [19] reported that HERV-K envelope protein was selectively expressed in cortical and spinal neurons of ALS patients and that the envelope protein was neurotoxic in stem-cell derived human neurons and in a transgenic mouse ALS model, strongly suggesting that HERV-K contributes to motor neuron disease.

In the present study we have attempted independent confirmation of elevated HERV-K transcripts in the cortex of ALS patients by using exactly the same *GAPDH* (Glyceraldehyde 3-phosphate dehydrogenase)-normalised RT-qPCR methods for quantification of HERV-K *gag, pol* and *env* transcripts as those described previously [19]. We have also repeated the quantification of cortical HERV-K transcripts using an alternative validated reference gene, *XPNPEP1* (X-prolyl aminopeptidase 1), as recommended by the MIQE guidelines [7]. Additionally, we investigated HERV-W, which has previously been associated with multiple sclerosis, schizophrenia and chronic inflammatory demyelinating polyneuropathy [17], in the same ALS and control samples by estimating its *env* RNA expression using a similar RT-qPCR method, to exclude it as a possible cause of the previously observed raised levels of serum reverse transcriptase in ALS.

## Materials and methods

### Clinical samples

Frozen postmortem brain material was obtained from the Medical Research Council (MRC) Neurodegenerative Disease Brain Bank Network, Institute of Psychiatry, Psychology and Neuroscience, Kings College London, UK. Premotor cortex (i.e. part of the frontal lobe just anterior to the primary motor cortex) from 34 patients with sporadic ALS and 23 non-ALS controls was analysed. Details including age, gender, diagnosis, postmortem delay and RNA integrity number (RIN) are presented in Additional file 1: Table S1. All ALS patients had their ALS clinical diagnosis confirmed by neuropathological examination of the brain postmortem.

### RNA extraction and quality control

60 mg pieces of frozen (− 80 °C) premotor cortex were homogenised (TissueRuptor II, Qiagen Ltd., Crawley, UK) on dry ice in QIAzol lysis buffer (Qiagen) and extracted using the RNeasy Lipid Tissue kit (Qiagen) with on-column DNase treatment (RNase-free DNase Set, Qiagen) according to the manufacturer’s instructions. RNA concentration was measured by Qubit™ RNA BR assay (Thermo Fisher Scientific Inc. Waltham, MA, USA) and RNA quality established by Agilent RNA 6000 Nano assay (Agilent Technologies, Inc. Santa Clara, CA, USA).

### cDNA synthesis

Reverse transcription of 1 μg of total RNA in a 20 μl reaction was performed using the Invitrogen SuperScript III First-Strand Synthesis Supermix for qRT-PCR (Thermo Fisher) according to manufacturer’s instructions. RNase H digestion was carried out at 37 °C for 20 mins and cDNA stored at − 20 °C. Negative control reverse transcription reactions without adding RNA were included in each batch of cDNA syntheses.

### HERV-K RT-qPCR

Real-time PCR was performed in an Applied Biosystems QuantStudio™ 5 thermocycler (Thermo Fisher), 96 well format, using Fast SYBR Green Master Mix (Thermo Fisher) in a 20 μl reaction with 2 μL cDNA and final concentrations of 0.25 μM forward primer and 0.25 μM reverse primer or 1X primer pool for *XPNPEP1*. Primer sequences for amplification of HERV-K *gag, pol* and *env*, and *GAPDH* were as described previously [19] and detailed in Table 1. Thermal cycling parameters were as follows: DNA polymerase activation, 95 °C for 20 s, then 45 cycles of denaturation, 95 °C for 1 s and anneal/extension 60 °C for 20 s. Cycling was followed by melt curve analysis. Automatic baseline settings were used with a manual threshold setting of 0.2 in all experiments. Each sample was analysed in duplicate and any sample with a Cq (quantification cycle) standard deviation of > 0.2 was repeated. All 96 well plates contained samples from both ALS patients and control individuals. No template controls (NTCs) were run in each experiment and every sample was analysed with and without reverse transcription in order to identify any residual genomic DNA contamination in the extracted RNA. Relative HERV-K RNA expression levels (i.e. relative with respect to the geometric mean of the non-ALS controls) were calculated using the 2^-∆∆Ct^ method [20] by normalisation against both *GAPDH* and *XPNPEP1* reference transcripts. RT-qPCR procedures were performed in accordance with the MIQE guidelines [7] (Additional file 1: Table S2) and all experiments were conducted ‘blind’ with the identity of each sample being hidden from the investigator. RT-qPCR experiments were conducted simultaneously on ALS cases and controls so as to eliminate such confounders as batch effects.

**Table 1:** Oligonucleotide sequence information

Primer name	Sequence 5′-3’	Reference
HERV-K *gag* forward^a^	AGCAGGTCAGGTGCCTGTAACATT	Li et al., [19]
HERV-K *gag* reverse	TGGTGCCGTAGGATTAAGTCTCCT	Li et al., [19]
HERV-K *pol* forward	TCACATGGAAACAGGCAAAA	Li et al., [19]
HERV-K *pol* reverse	AGGTACATGCGTGACATCCA	Li et al., [19]
HERV-K *env* forward	CTGAGGCAATTGCAGGAGTT	Li et al., [19]
HERV-K *env* reverse	GCTGTCTCTTCGGAGCTGTT	Li et al., [19]
*GAPDH* forward	TGCACCACCAACTGCTTAGC	Li et al., [19]
*GAPDH* reverse	GGCATGGACTGTGGTCATGAG	Li et al., [19]
*XPNPEP1* forward	Qiagen cat no. QT00051471	Qiagen^a^
*XPNPEP1* reverse	Qiagen cat no. QT00051471	Qiagen^a^
HERV-W *env* forward	GTATGTCTGATGGGGGTGGAG	Levet et al., [18]
HERV-W *env* reverse	CTAGTCCTTTGTAGGGGCTAGAG	Levet et al., [18]

^a^All primers were synthesised by Eurofins Genomics (Germany) apart from the *XPNPEP1* primers which were obtained from Qiagen (Hs_*XPNPEP1*_1_SG QuantiTect Primer. Product number: 249900. Cat no: QT00051471)

### HERV-W RT-qPCR

HERV-W *env* RNA was quantified in all samples by the same method used for HERV-K RNA quantification but using the HERV-W *env* specific primers (Table 1) described previously [18].

### Reference gene validation

A panel of 9 candidate reference gene primer sets were evaluated in order to establish the one with the most stable level of expression in ALS and non-ALS control premotor cortex samples. Briefly, RT-qPCR was used to quantify the RNA expression levels of *GAPDH, ACTB, CYC1, SDHA, UBC, RPL13A, XPNPEP1, EIF4A2* and *YWHAZ* in 5 ALS samples and 5 non-ALS control samples in triplicate. All primer sets apart from *XPNPEP1* were obtained from Primerdesign Ltd., Camberley, UK. The evaluation was conducted using qBase+ software, version 3.1 (Biogazelle, Zwijnaarde, Belgium), which utilises the geNorm selection algorithm [31], along with additional statistical tools. Further verification was performed using RefFinder software [33] which exploits the computational programs Normfinder [2], BestKeeper [27], geNorm and the ΔCt method [28] to comprehensively rank and compare candidate reference genes.

### Sequencing

Amplicons generated by the HERV-K *gag,* HERV-K *pol,* HERV-K *env,* HERV-W *env, GAPDH* and *XPNPEP1* PCRs were subjected to Sanger dideoxy sequencing (Eurofins GTAC Biotech, Germany) and sequences analysed by BLAST (Basic Local Alignment Search Tool) in order to check the specificity of each assay. In each case amplicon size was confirmed by agarose gel electrophoresis.

### Statistical analysis

The statistical significance of differences between groups was assessed by 2-tailed Mann-Whitney U test (GraphPad Prism 7 software). *p*-values of < 0.05 were considered significant. Linear regression *p*-values were calculated using Microsoft Excel Add-In Daniel’s XL toolbox v6.22.

## Results

### Matching of ALS patients with non-ALS control group

Due to limited availability of suitable postmortem material it was not always possible to obtain a perfect match of all clinical parameters between the ALS patient group and the non-ALS control group. The mean age at death was 66.9 years for the ALS patients and 73.5 years for the controls (*p* = 0.03). There was also a difference in gender distribution between the ALS and control groups. The ALS group was 29% female whereas the control group was 48% female. There was no significant difference in mean postmortem delay between the patients and controls; for the ALS group it was 45.1 h and for the control group 41.7 h (*p* = 0.64). Finally, there was a small difference between the mean RNA integrity value (RIN) of the ALS samples, 6.53, and that of the controls, 6.05 (*p* = 0.01). The potential effect of such imperfectly matched parameters on HERV-K expression comparisons between ALS patients and controls is considered below.

### RT-qPCR performance characteristics

Agarose gel electrophoresis confirmed that the amplicons generated by each of the six different RT-qPCR assays (HERV-K *gag*, HERV-K *pol*, HERV-K *env*, HERV-W *env*, *GAPDH*, *XPNPEP1*) were single bands of the expected size. Melt curve analysis also revealed single dominant peaks for all assays. Amplification specificity was further confirmed by Sanger sequencing of the amplicons which demonstrated that the nucleotide sequence was as expected for all six RT-qPCR assays (Basic Local Alignment Search Tool, BLASTn, analysis). PCR efficiencies for each assay were estimated from slopes of serial dilution standard curves using the formula E = 10^(− 1/slope) – 1 and all fell within the range 99% ± 6%. For each assay the R^2^ value of the standard curve was > 0.99. The no template controls were negative in all experimental runs and the no reverse transcriptase controls revealed that residual genomic DNA in RNA extracts typically contributed less than 1%, but always less than 3%, of the total signal in all samples.

### Reference gene validation

qBase+ and RefFinder ranking of the nine candidate reference genes revealed that *XPNPEP1* and *GAPDH* had the most stable expression in ALS and control samples (Additional file 1: Table S3). *XPNPEP1* was therefore selected as the additional ‘validated’ reference gene to use in addition to *GAPDH* for the normalisation of HERV-K and HERV-W RNA expression levels.

### HERV-K RNA expression in ALS and controls

**Fig. 1:**
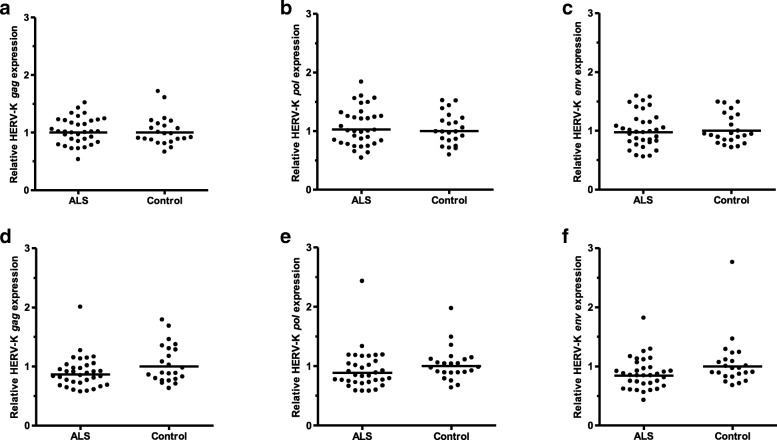
Relative expression levels of HERV-K *gag, pol* and *env* RNA in ALS and non-ALS controls. **a**, **b**, **c** normalised against *GAPDH*; **d**, **e**, **f** normalised against *XPNPEP1.* Horizontal black lines represent geometric means. All *p* values are > 0.05, NS

**Fig. 2:**
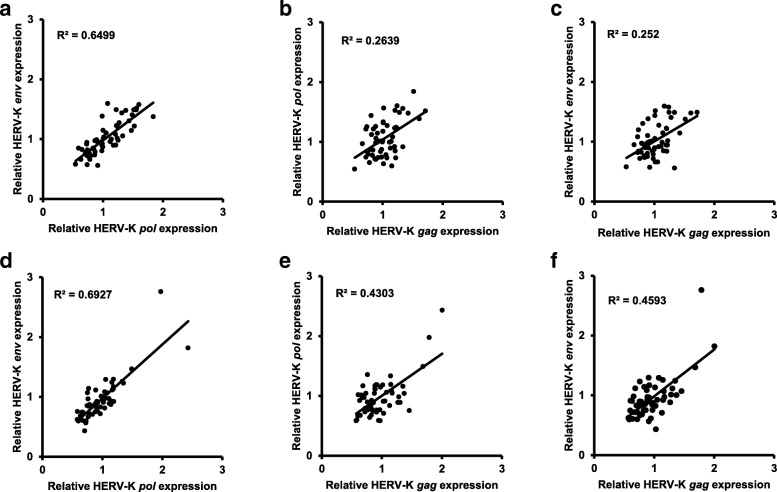
Correlations between HERV-K *gag, pol* and *env* RNA relative expression levels. Data from all 34 ALS and 23 non-ALS controls are presented. **a**, **b**, **c** normalised against *GAPDH*; **d**, **e**, **f** normalised against *XPNPEP1.* R-squared coefficient of determination values calculated in Microsoft Excel. All p values are < 0.0001 by linear regression

Figure 1 and Table 2 summarise the relative expression levels of HERV-K *gag, pol* and *env* RNA in the 34 ALS cases and 23 non-ALS controls investigated. When the *GAPDH* reference gene was used for normalisation, the geometric mean expression levels for HERV-K *gag* and HERV-K *pol* were very slightly higher in the ALS patients than in the control group but the converse was true for HERV-K *env*. None of these marginal differences in HERV-K expression levels approached statistical significance. When the *XPNPEP1* reference gene was used for normalisation the geometric mean expression levels for all three HERV-K genes were slightly lower in ALS cases than in controls. Once again these small differences in geometric mean expression levels failed to reach statistical significance. However, the expression levels of HERV-K *gag*, *pol* and *env* were correlated with each other whether the data was normalised by *GAPDH* or *XPNPEP1* reference genes (Fig. 2).

**Table 2:** Geometric mean relative expression of HERV-K RNA in ALS cases and controls

	HERV-K *gag*	HERV-K *pol*	HERV-K *env*	HERV-K *gag*	HERV-K *pol*	HERV-K *env*
Normalisation method^a^	*GAPDH*	*GAPDH*	*GAPDH*	*XPNPEP1*	*XPNPEP1*	*XPNPEP1*
ALS	1.001	1.025	0.975	0.868	0.888	0.845
Control	1	1	1	1	1	1
p value	0.677	0.671	0.990	0.113	0.095	0.055
Statistical significance	NS^b^	NS	NS	NS	NS	NS

^a^ Normalisation against either *GAPDH* or *XPNPEP1* reference genes

^b^ NS = the difference was not statistically significant at *p* < 0.05

As perfect matching between the ALS patients and non-ALS controls of parameters such as age, gender, postmortem delay and RIN values was not always possible due to limited availability of suitable postmortem material, these variables were also examined for possible correlation with HERV-K expression. Neither age nor gender was correlated with HERV-K *gag*, *pol* or *env* expression levels whether data was normalised by *GAPDH* or *XPNPEP1* reference genes (Additional file 1: Figure S1 and Figure S2). A trend towards reduced HERV-K RNA levels with increased postmortem delay was observed (Additional file 1: Figure S3) for HERV-K *pol* and *env,* whether normalisation was to *GAPDH* or *XPNPEP1* reference genes (*p* ≤ 0.01). RIN values were not correlated with HERV-K *gag, pol* or *env* RNA levels when *GAPDH* normalisation was used but there was a slight negative correlation between high RIN values and *XPNPEP1*-normalised relative HERV-K *gag* and *env* RNA levels (*gag*, *p* = 0.04 and *env*, *p* = 0.03, Additional file 1: Figure S4).

### HERV-W *env* RNA expression in ALS and controls

**Fig. 3:**
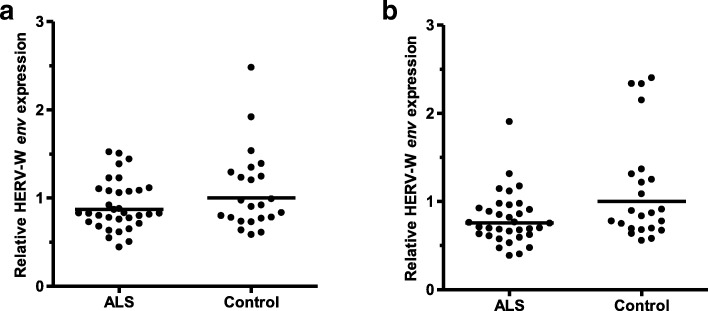
Relative expression levels of HERV-W *env* RNA in 34 ALS and 23 non-ALS controls. (**a**) normalised against *GAPDH*; (**b**) normalised against *XPNPEP1*. *P* value for the difference between the groups when normalised by *GAPDH* was *p* = 0.26 and when normalised by *XPNPEP1* was *p* = 0.04. Horizontal black lines represent geometric means

Figure 3 shows the relative HERV-W *env* RNA expression levels in ALS cases and non-ALS controls. When *GAPDH* was employed for normalisation, the geometric mean HERV-W *env* RNA level for the ALS cases was 0.87 and for the controls 1.00 (*p* = 0.26). However, when *XPNPEP1* was used for normalisation the geometric mean HERV-W *env* RNA level in ALS was lower at 0.75 than in controls at 1.00 (*p* = 0.04).

HERV-W *env* RNA levels showed no correlation with age or gender whether normalised by *GAPDH* or *XPNPEP1* (Additional file 1: Figure S5 and Figure S6). Postmortem delay was not significantly correlated with HERV-W *env* RNA levels when *XPNPEP1* normalisation was used but there was a downward trend of HERV-W *env* RNA level with increased PMD when *GAPDH* normalisation was used (p = 0.04) (Additional file 1: Figure S7). With *XPNPEP1* normalisation, high RIN values were correlated with low relative expression of HERV-W *env* RNA (*p* = 0.001) but there was no correlation when *GAPDH* normalisation was used (Additional file 1: Figure S8).

### Correlation between *GAPDH*-normalised and *XPNPEP1*-normalised expression data

*GAPDH*-normalised relative expression levels and *XPNPEP1*-normalised relative expression levels correlated well with each other for HERV-K *gag, pol* and *env*, and also for HERV-W *env* transcripts (Additional file 1: Figure S9).

## Discussion

Overexpression of HERV-K has recently been proposed as a possible causative factor in patients with ALS [19]. Although this is an attractive hypothesis, not least because it raises the possibility that ALS might become treatable using antiretroviral drugs [26] or antibodies [13], the contribution of HERV-K to the disease process has yet to be conclusively proven. The magnitude of the difference reported previously [19] between the mean HERV-K RNA expression level in ALS patients and non-ALS controls was less than threefold for *gag* and *env*, and less than two fold for *pol*. Although such relatively modest differences can in principle be resolved by RT-qPCR due to the high technical precision of the method, it is essential to demonstrate their reproducibility in other patient cohorts by independent testing such as that undertaken here. In this study we have therefore attempted confirmation of the observation that *GAPDH*-normalised HERV-K RNA levels are elevated in the cerebral cortex of ALS patients with respect to non-ALS controls. Postmortem premotor cortex samples from 34 patients with ALS and 23 non-ALS controls were tested using the same RT-qPCR methods (including the same reverse transcription method, the same DNase treatment method, the same PCR reagents and the same thermal cycling parameters), the same 2^-∆∆Ct^ data analysis method and identical primer sets to those used previously [19]. Our observations were concordant with previous findings [19] regarding the good correlation between the relative expression levels of the three HERV-K transcripts *gag, pol* and *env,* suggesting that the entire viral genome was expressed. However, in contrast to the findings of Li et al. [19], we were unable to demonstrate any difference in HERV-K *gag, pol* or *env* RNA levels between ALS patients and controls, whether the data were normalised by *GAPDH* or *XPNPEP1* reference genes.

The reasons for this conflict are uncertain but several possibilities exist. Firstly, our ALS patients and non-ALS controls were not perfectly matched for age, gender, postmortem delay or RIN values and the differences between the groups were statistically significant for age, gender and RIN. However, none of these three parameters was found to be correlated with *GAPDH*-normalised HERV-K RNA expression and so their imperfect matching does not appear to explain why our results differ from those published previously [19]. Secondly, it is conceivable that differences between the USA ALS cohort [19] and our UK ALS cohort might explain the discrepant results. We consider this unlikely because the diagnosis of ALS was confirmed by neuropathological examination in both cohorts and tissue samples in the two cohorts were derived from similar neuroanatomical regions. Nevertheless, it is theoretically possible that there is a small subset of ALS patients who do have elevated cortical HERV-K expression and that members of that subset were present in the ALS cohort studied by Li et al. but not in our cohort. However, in comparison with our ALS samples, the mean postmortem delay was shorter and the mean RIN value was higher in the samples analysed previously [19]. Although we found no significant correlation between *GAPDH*-normalised HERV-K RNA expression and RIN we did observe a trend towards lower *GAPDH*-normalised HERV-K RNA levels (*pol* and *env* only) with increasing postmortem delay. Nevertheless, we consider it unlikely that this difference in mean postmortem delay explains our failure to confirm the previously published findings [19] because the HERV-K RNA RT-qPCR quantitative data is not ‘absolute’ but ‘relative’ with respect to the geometric mean level of the non-ALS controls which had postmortem delays not significantly different from the ALS cases. In agreement with a previous report on human postmortem brain RNA quality [11] we found no correlation between RIN value and postmortem delay (Additional file 1: Figure S10). Thirdly, it is conceivable that the discrepancy may be related to differences in the proportion of controls with cancer. Twenty five percent of the control group used by Li et al. had cancer as against 47% in the present study. Since upregulation of HERV-K expression occurs in various types of tumour tissue [30] it could be argued that there is a remote possibility that HERV-K expression could be elevated in the brains of the controls who had cancer, even without cerebral metastases. However, this explanation can be dismissed because our conclusion that patients with ALS do not have significantly higher levels of cerebral HERV-K RNA expression than non-ALS controls remains unchanged on reanalysing the data following exclusion of those with cancer from the control group (Additional file 1: Figure S11). Finally, the relatively low number of ALS cases studied by Li et al. (*n* = 11) may have resulted in less statistically robust findings than those generated by our larger analysis of 34 cases.

For ensuring accurate quantification, the importance of selecting appropriate reference genes for normalisation in RNA expression studies has been stressed by many authors, as has the advantage of using more than one reference gene [7, 15]. In the present study we evaluated a panel of nine candidate reference genes including *GAPDH* which had been used previously [19]. This evaluation revealed that *XPNPEP1* and *GAPDH* had the most stable expression in ALS and non-ALS control material, and *XPNPEP1* was therefore chosen as the additional reference gene. It is noteworthy that Durrenberger et al. [10] in an evaluation of 12 candidate reference genes for use on human CNS postmortem tissue in various neurological diseases including ALS, also identified *XPNPEP1* as the most stable and suitable candidate. *GAPDH*-normalised relative expression levels and *XPNPEP1*-normalised relative expression levels were generally well correlated. Our conclusion that HERV-K RNA levels are not elevated in the premotor cortex of ALS patients with respect to non-ALS controls was the same whichever reference gene was used for data normalisation.

In the study by Li et al. [19], cerebral cortical expression of a number of other HERVs, in addition to HERV-K, was measured. They examined HERV-E, HERV-R and HERV-P by RT-qPCR but did not observe significant ALS-associated elevation of transcripts in any of these. We considered that HERV-W should also be investigated because it had been implicated in a number of other neurological conditions including multiple sclerosis, schizophrenia and chronic inflammatory demyelinating polyneuropathy [17]. Although our study does not show any association between HERV-W RNA expression level and ALS when data is *GAPDH*-normalised, a small negative correlation (*p* = 0.04) between HERV-W RNA expression and ALS was observed with *XPNPEP1* normalisation due in part to four control samples showing relatively high HERV-W *env* expression when normalised to this reference gene. We speculate however that this small negative correlation may be unreliable due to our ALS samples having a slightly higher mean RIN value than controls, in conjunction with high RIN values being significantly correlated with lower *XPNPEP1*-normalised relative HERV-W expression.

Another study investigating the potential role of HERV-K in ALS has just been published by Mayer et al. [24]. Using an alternative RT-qPCR method they examined levels of *GAPDH*-normalised HERV-K *gag* RNA in frozen postmortem neural tissues from a total of 108 ALS and control samples obtained from brain banks in the USA. As in our study, Mayer and colleagues were unable to confirm the previous findings [19] and concluded that levels of HERV-K transcripts were not significantly different between ALS and controls. Furthermore, they were unable to demonstrate any significant difference between ALS and controls in the pattern of transcriptionally active HERV-K loci by sequencing-based transcriptional profiling, or indeed any evidence of full length HERV-K envelope protein in either ALS or control tissues. These transcriptional profiling results also conflict with the findings of Li et al. [19] who observed three HERV-K loci to be transcribed at higher levels in ALS cases than in controls. However, it is important to note that the failure of our study and that of Mayer et al. to confirm elevated levels of HERV-K RNA in ALS post-mortem brain tissue does not diminish the significance of the report by Li et al. [19] that expression of HERV-K in human neurons in vitro causes toxicity, or that expression of HERV-K env in transgenic mice causes degeneration of motor neurons and progressive motor dysfunction.

## Conclusion

In conclusion, our observations and those recently published by Mayer and colleagues [24] fail to confirm the findings of Li et al. [19] and provide no support for the hypothesis that elevated HERV-K expression in the cerebral cortex is associated with sporadic amyotrophic lateral sclerosis. These conflicting results may have significant implications for ALS clinical trials aiming to suppress HERV-K activity with antiretroviral drugs, and suggest that further research in this area is required to discover the source of the increased serum reverse transcriptase activity seen in this disease. Future studies that we propose include the use of next generation sequencing and custom-made microarrays to undertake a broad screening of the expression profiles of all human endogenous retrovirus families in ALS and controls.

## Additional file

Additional file 1: **Figures S1.**–**Figure S11.** and **Tables S1**–**Table S3.** (PDF 874 kb)

## Abbreviations

ALS: Amyotrophic lateral sclerosis
Cq: quantification cycle (also referred to as Ct or threshold cycle)
*GAPDH*: Glyceraldehyde 3-phosphate dehydrogenase
HERV-K: human endogenous retrovirus-K (HML-2 subfamily)
HERV-W: human endogenous retrovirus-W
MIQE guidelines: Minimum Information for Publication of Quantitative Real-Time PCR Experiments
PMD: postmortem delay
RIN: RNA integrity number
RT-qPCR: quantitative real-time reverse transcription polymerase chain reaction
*XPNPEP1*: X-prolyl aminopeptidase
