# Supplementary material for: Quantitative analysis of human endogenous retrovirus-K transcripts in postmortem premotor cortex fails to confirm elevated expression of HERV-K RNA in amyotrophic lateral sclerosis
Source: Acta Neuropathol Commun. 2019 Mar 18;7:45. doi: 10.1186/s40478-019-0698-2 (PMC6421708; doi:10.1186/s40478-019-0698-2)
Supplement: Supplementary file 1 — Figures S1.–Figure S11. and Tables S1–Table S3. (PDF 874 kb) [file 40478_2019_698_MOESM1_ESM.pdf]

Quantitative analysis of Human Endogenous Retrovirus-K transcripts in postmortem premotor cortex fails to confirm elevated expression of HERV-K RNA in amyotrophic lateral sclerosis

Jeremy A. Garson[1,2], Louise Usher[3], Ammar Al-Chalabi[4], Jim Huggett[5,6], Edmund F. Day[3], Adele L. McCormick[3]

1. Division of Infection and Immunity, University College London, London, UK
2. National Transfusion Microbiology Laboratories, NHS Blood and Transplant, Colindale, London, UK
3. School of Life Sciences, University of Westminster, London, UK
4. Maurice Wohl Clinical Neuroscience Institute, King's College London, London, UK
5. Molecular and Cell Biology Team, LGC, Teddington, UK
6. School of Biosciences and Medicine, Faculty of Health and Medical Science, University of Surrey, Guildford, UK

Corresponding author: J A Garson, email: j.garson@ucl.ac.uk

CONTENTS

**Supplementary Figures**	**Page**
Figure S1	2
Figure S2	3
Figure S3	4
Figure S4	5
Figure S5	6
Figure S6	6
Figure S7	7
Figure S8	7
Figure S9	8
Figure S10	8
Figure S11	9
**Supplementary Tables**	
Table S1 (Clinical details of ALS cases and controls)	10
Table S2 (MIQE checklist)	11
Table S3 (RefFinder rankings of candidate reference genes)	14

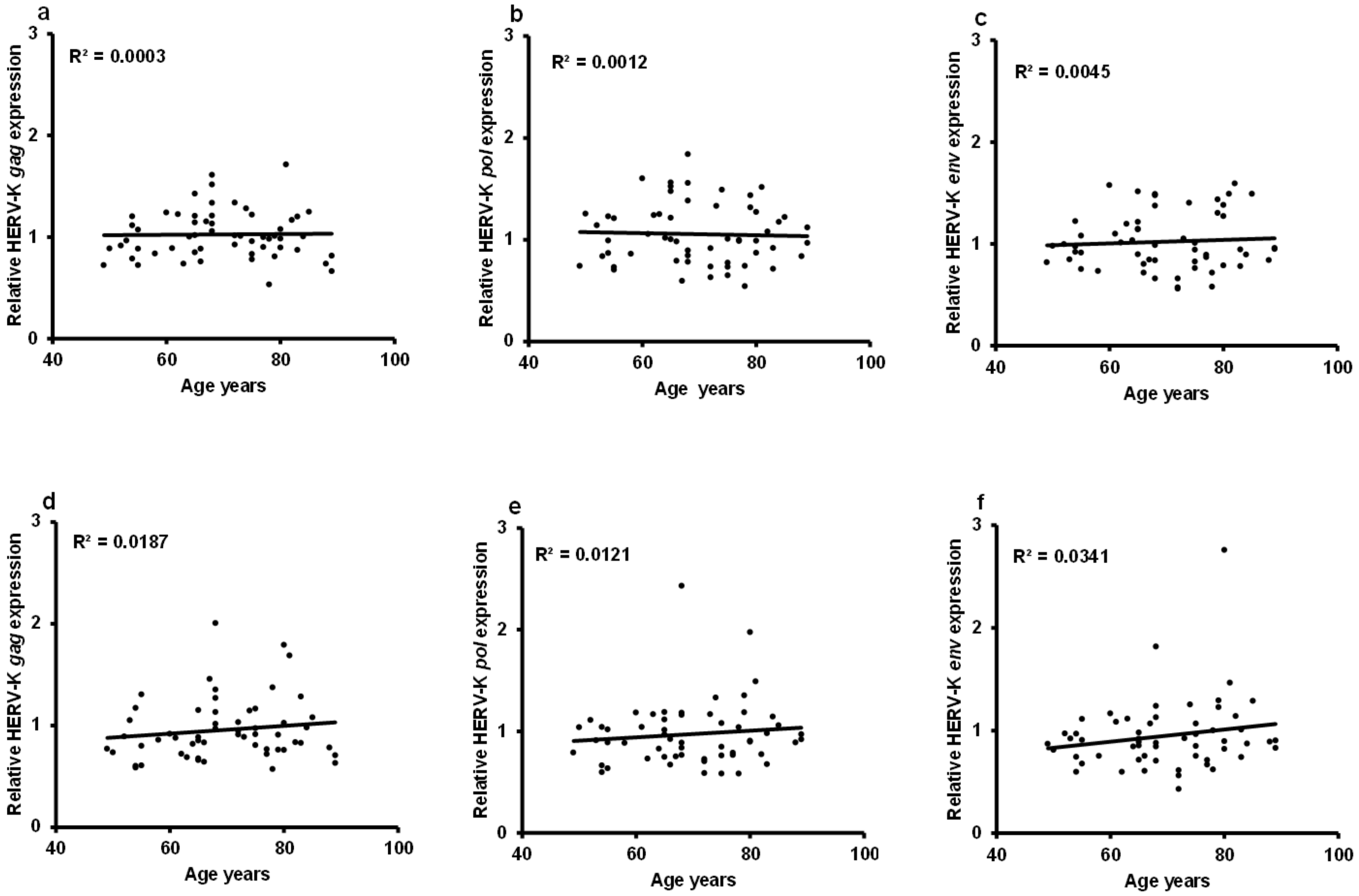

Additional file1: Figure S1. No significant correlation between age and HERV-K *gag, pol* and *env* RNA relative expression levels whether normalised by *GAPDH* or *XPNPEP1*. Data from all 34 ALS and 23 non-ALS controls are presented. **a, b, c** normalised against *GAPDH*; **d, e, f** normalised against *XPNPEP1.* R-squared coefficient of determination values calculated in Microsoft Excel. All p values are >0.05 by linear regression, NS.

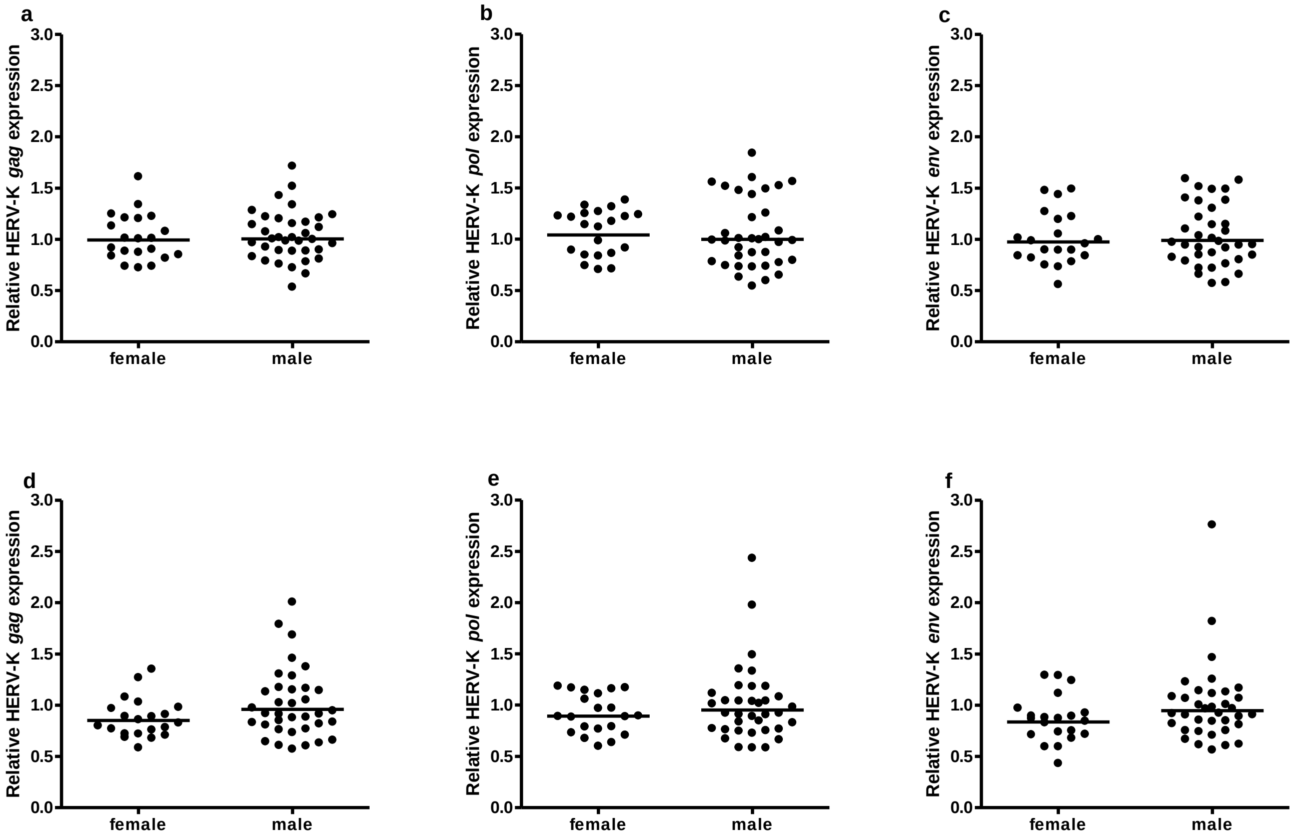

Additional file1: Figure S2. No significant correlation between gender and HERV-K RNA expression whether normalised by *GAPDH* or *XPNPEP1*. Data from all 34 ALS and 23 non-ALS controls are presented. **a, b, c** normalised against *GAPDH*; **d, e, f** normalised against *XPNPEP1.* All p values are >0.05, NS. Horizontal black lines represent geometric means

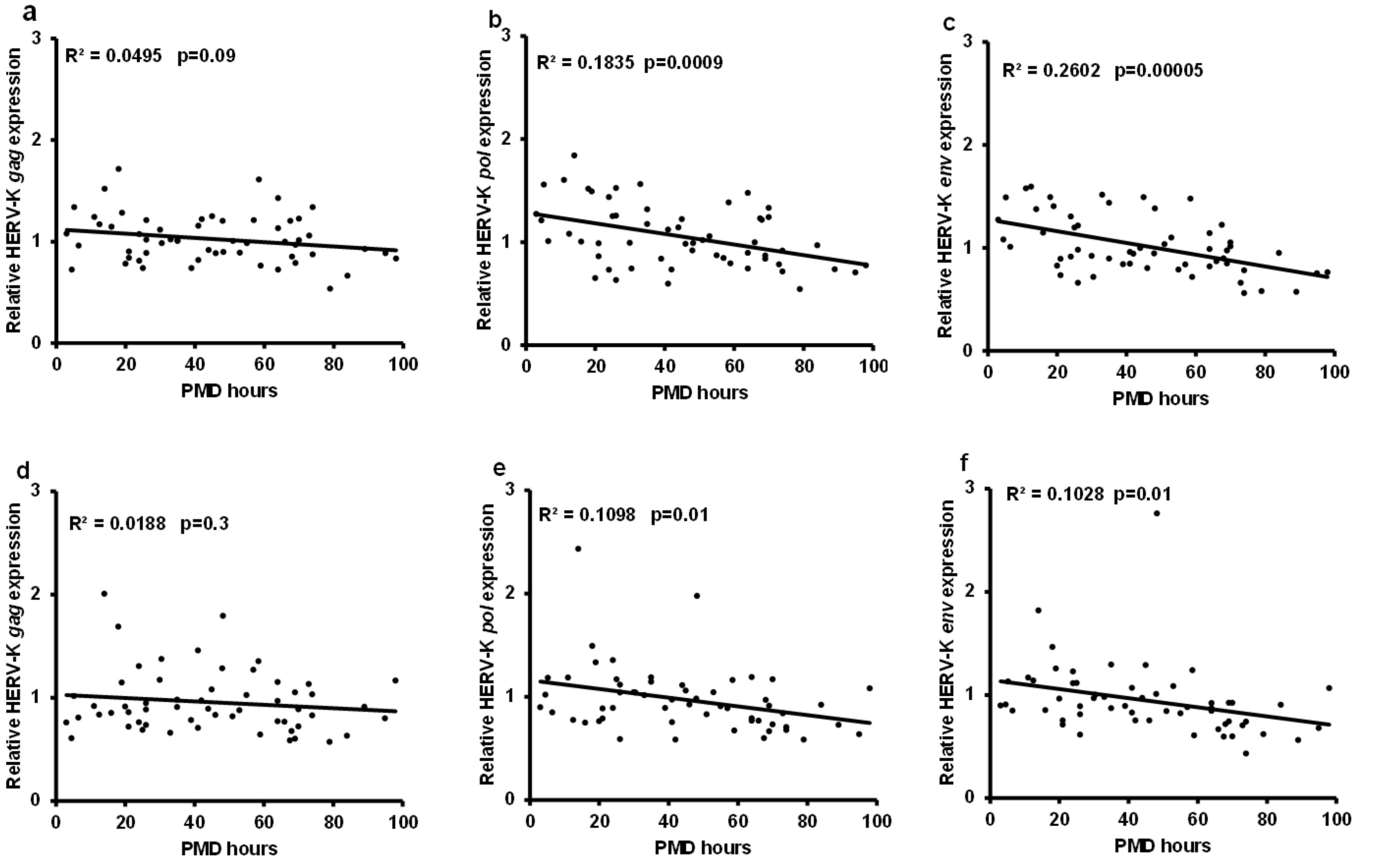

Additional file1: Figure S3. Correlation between high postmortem delay (PMD) and reduced relative HERV-K RNA expression reaches statistical significance for *pol* and *env* whether normalised by *GAPDH* or *XPNPEP1.* Data from all 34 ALS and 23 non-ALS controls are presented. **a, b, c** normalised against *GAPDH*; **d, e, f** normalised against *XPNPEP1.* R-squared coefficient of determination values calculated in Microsoft Excel. Linear regression p values are shown in each graph

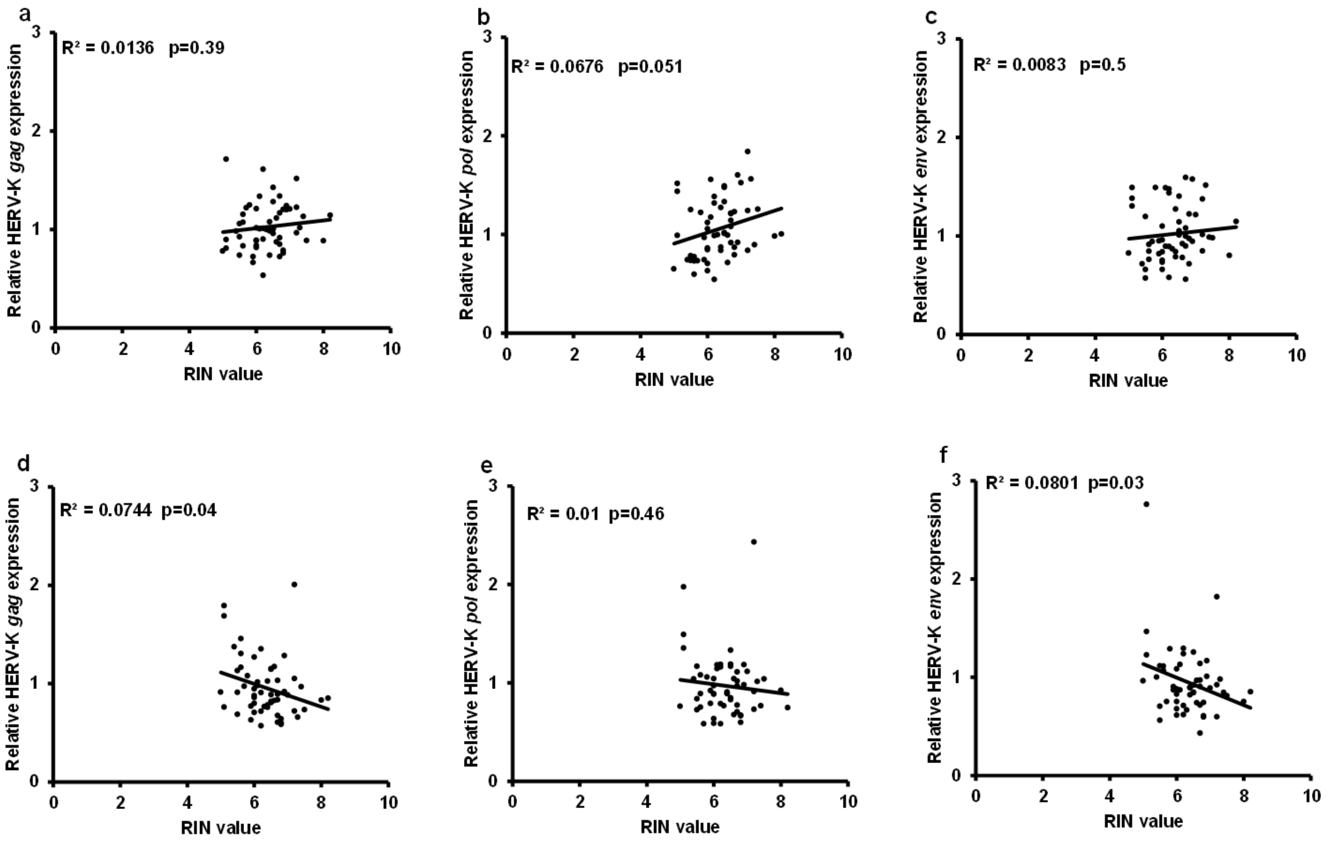

Additional file1: Figure S4. Correlation between RNA integrity number (RIN) and HERV-K *gag, pol* and *env* RNA relative expression levels. Data from all 34 ALS and 23 non-ALS controls are presented. **a, b, c** normalised against *GAPDH*; **d, e, f** normalised against *XPNPEP1.* R-squared coefficient of determination values calculated in Microsoft Excel. Linear regression p values are shown in each graph

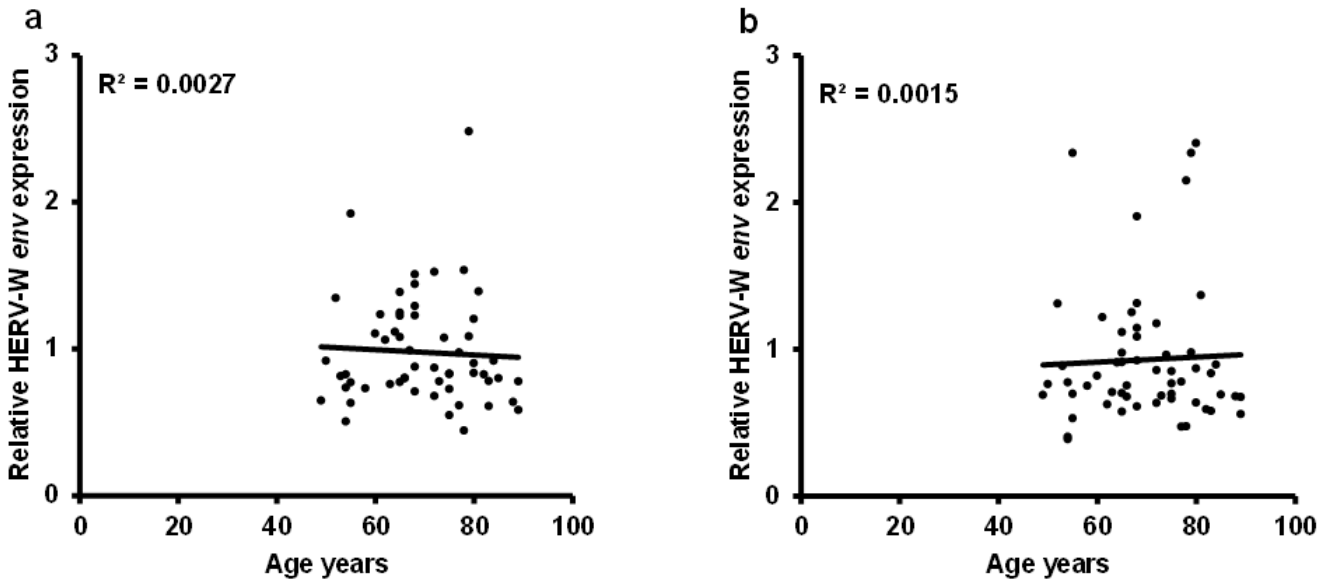

Additional file1: Figure S5. HERV-W *env* RNA relative expression shows no significant correlation with age. Data from all 34 ALS and 23 non-ALS controls are presented. **a**, *GAPDH* normalised, **b** *XPNPEP1* normalised. R-squared coefficient of determination values calculated in Microsoft Excel. Both p values are >0.05 by linear regression, NS.

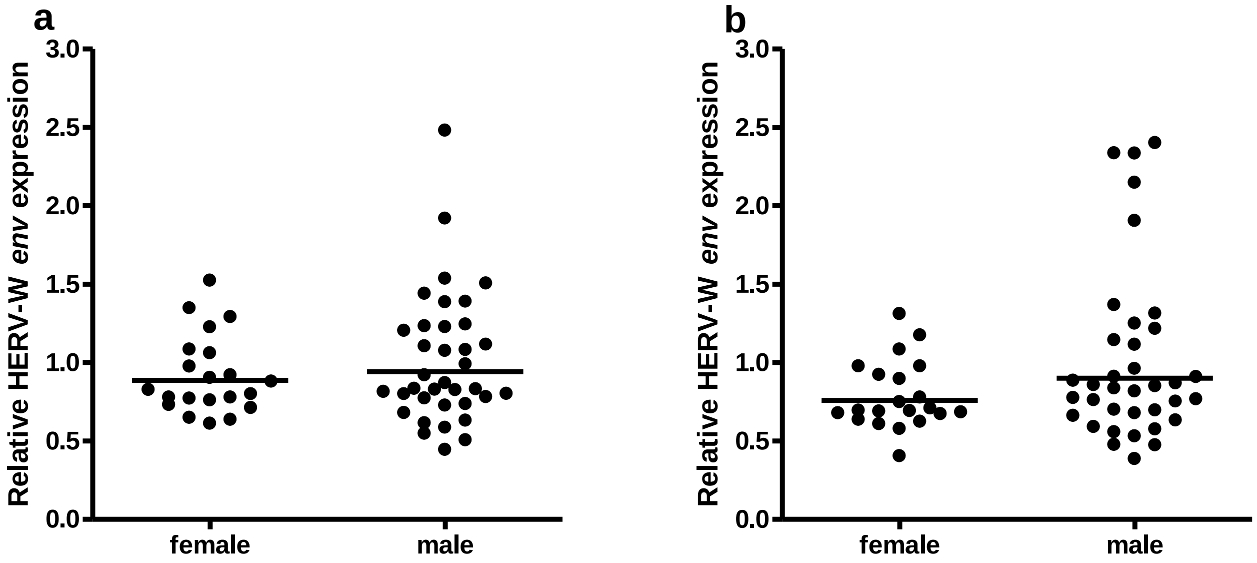

Additional file1: Figure S6. HERV-W *env* RNA relative expression shows no significant correlation with gender. Data from all 34 ALS and 23 non-ALS controls are presented. **a**, *GAPDH* normalised, **b** *XPNPEP1* normalised. Both p values are >0.05, NS. Horizontal black lines represent geometric means

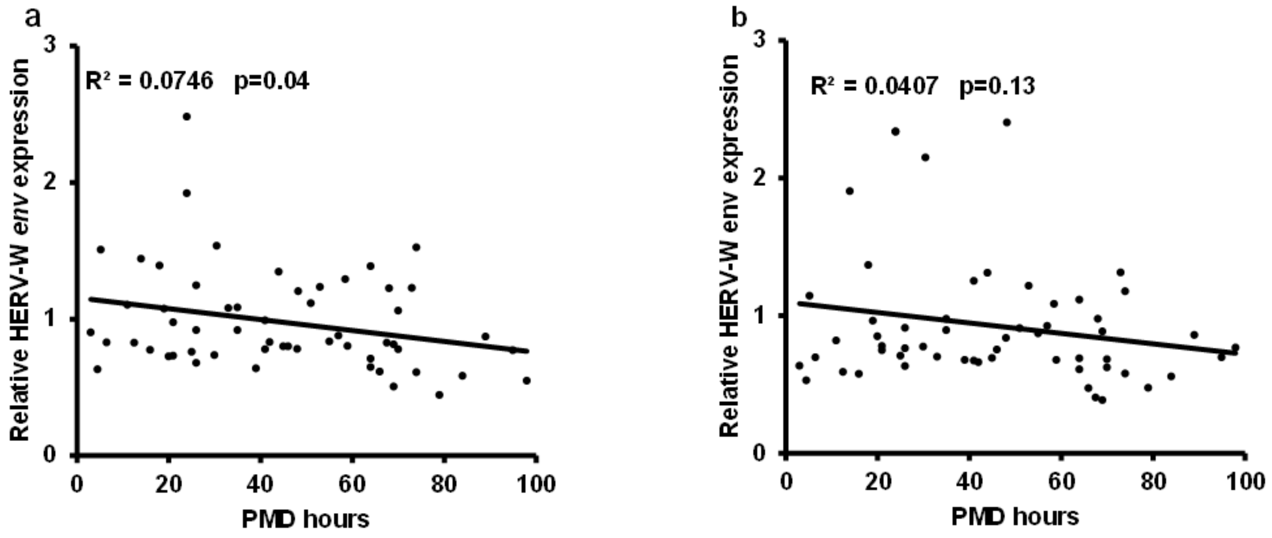

Additional file1: Figure S7. HERV-W *env* RNA relative expression correlated with postmortem delay (PMD). Data from all 34 ALS and 23 non-ALS controls are presented. **a**, *GAPDH* normalised, **b** *XPNPEP1* normalised. R-squared coefficient of determination values calculated in Microsoft Excel. Linear regression p values are shown in each graph

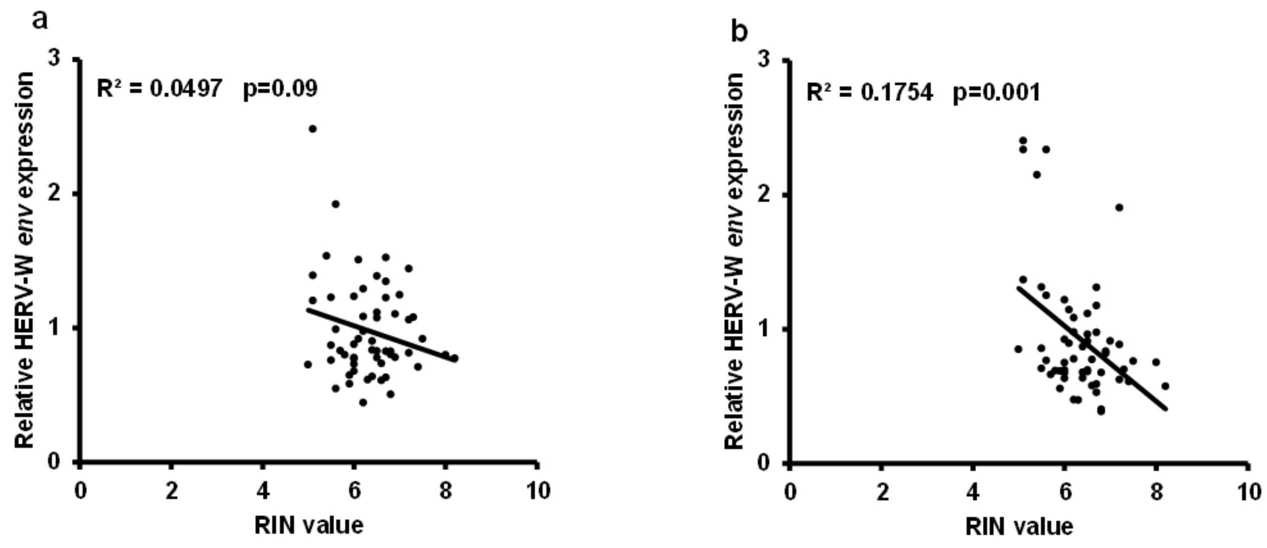

Additional file1: Figure S8. HERV-W *env* RNA relative expression correlated with RNA integrity number (RIN). Data from all 34 ALS and 23 non-ALS controls are presented. **a**, *GAPDH* normalised, **b** *XPNPEP1* normalised. R-squared coefficient of determination values calculated in Microsoft Excel. Linear regression p values are shown in each graph

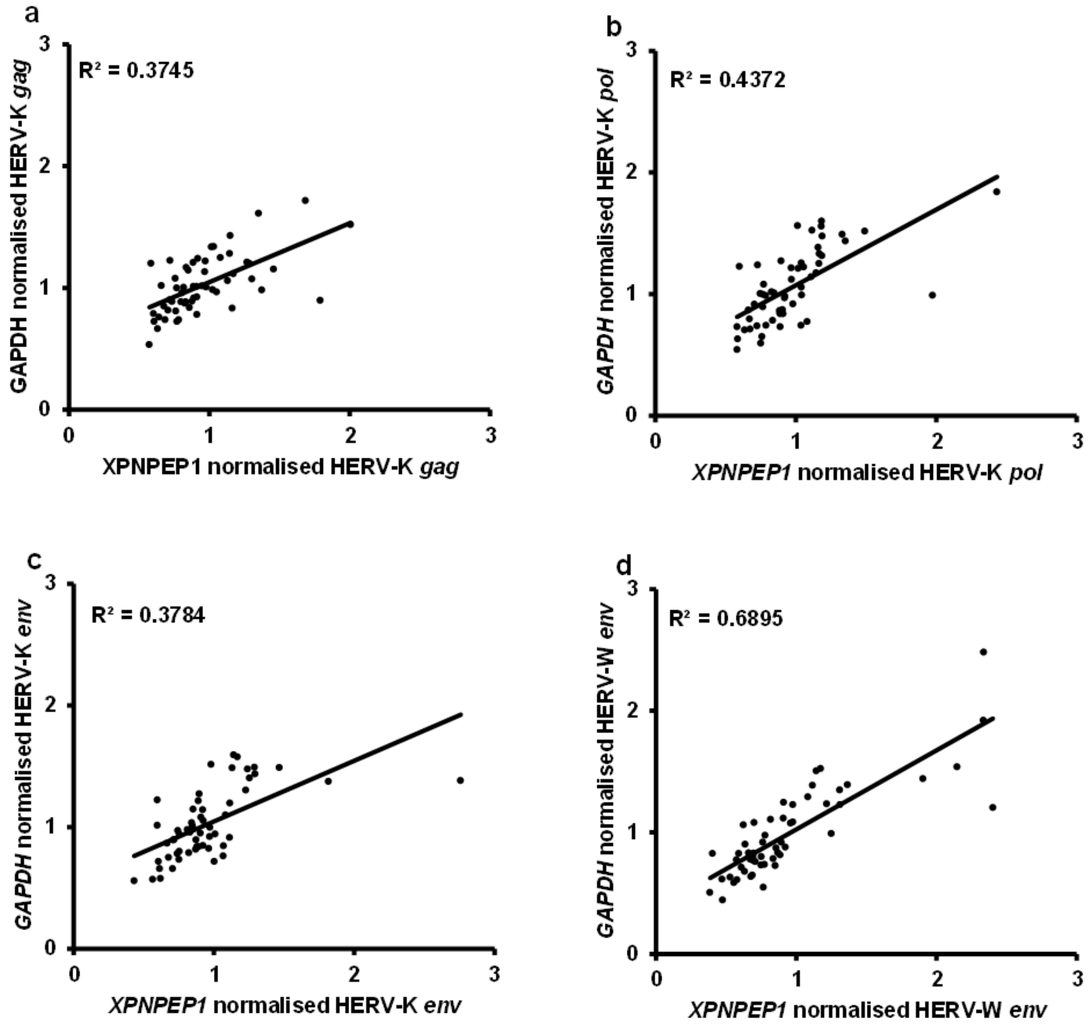

Additional file1: Figure S9. Correlation between *GAPDH* and *XPNPEP1* normalised HERV-K and HERV-W RNA relative expression levels. Parts **a**, **b** and **c** represent HERV-K *gag, pol* and *env* respectively. Part **d** represents HERV-W *env*. Data from all 34 ALS and 23 non-ALS controls are presented. R-squared coefficient of determination values calculated in Microsoft Excel. All p values are <0.000001 by linear regression

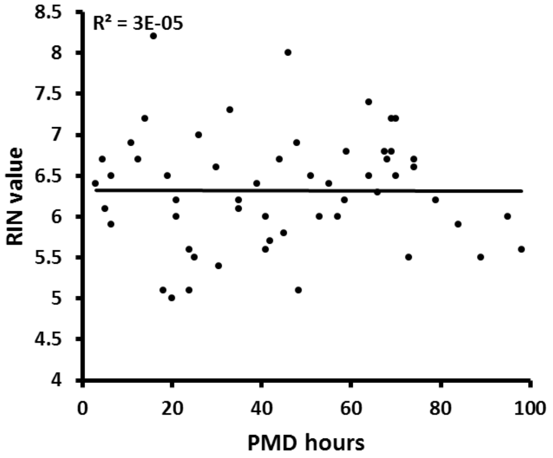

Additional file1: Figure S10. No correlation between RNA integrity number (RIN) and post mortem delay (PMD). Data from all 34 ALS and 23 non-ALS controls are presented. R-squared coefficient of determination values calculated in Microsoft Excel.

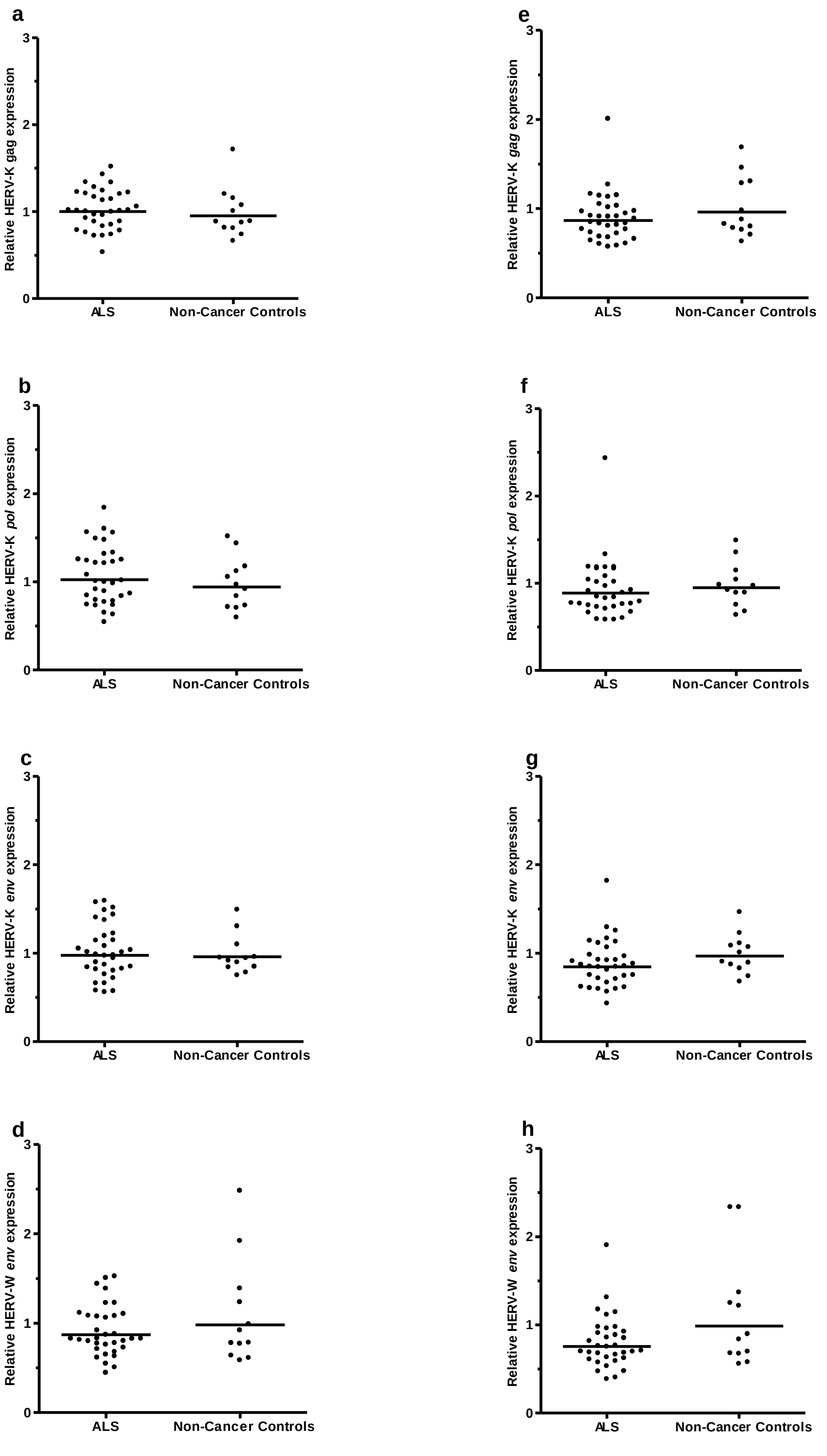

Additional file1: Figure S11. Relative expression levels of HERV-K *gag, pol* and *env* RNA, and HERV-W *env* RNA in 34 ALS and 12 non-ALS controls without cancer. **a, b, c** and **d** normalised against *GAPDH*; **e, f, g** and **h** normalised against *XPNPEP1.* Horizontal black lines represent geometric means. All p values are >0.05, (no significant difference) varying between 0.15 and 0.77

Additional file1: Table S1. Clinical details of ALS cases and controls

Sample no.	Diagnosis/cause of death	Gender	Age (years)	PMD[a] (hours)	RIN[b]
ALS #1	ALS[c]	F	54	67.5	6.8
ALS #2	ALS	F	65	68	6.7
ALS #3	ALS	F	73	70	6.5
ALS #4	ALS	M	65	16	8.2
ALS #5	ALS	F	62	70	7.2
ALS #6	ALS	F	68	64	7.4
ALS #7	ALS	M	78	79	6.2
ALS #8	ALS	M	77	66	6.3
ALS #9	ALS	M	66	46	8.0
ALS #10	ALS	M	65	33	7.3
ALS #11	ALS	M	65	64	6.5
ALS #12	ALS	F	68	57	6.0
ALS #13	ALS	F	63	25	5.5
ALS #14	ALS	M	68	73	5.5
ALS #15	ALS	F	72	74	6.7
ALS #16	ALS	M	75	42	5.7
ALS #17	ALS	M	64	51	6.5
ALS #18	ALS	M	53	69	7.2
ALS #19	ALS	M	66	59	6.8
ALS #20	ALS	M	72	89	5.5
ALS #21	ALS	M	72	26	6.0
ALS #22	ALS	M	74	19	6.5
ALS #23	ALS	M	75	20	5.0
ALS #24	ALS	M	82	12.5	6.7
ALS #25	ALS	M	75	6.5	6.5
ALS #26	ALS	M	68	14	7.2
ALS #27	ALS	F	79	35	6.2
ALS #28	ALS	M	75	98	5.6
ALS #29	ALS	M	68	5.2	6.1
ALS #30	ALS	M	55	4.5	6.7
ALS #31	ALS	M	60	11	6.9
ALS #32	ALS	F	49	64	5.9
ALS #33	ALS	M	50	26	7.5
ALS #34	ALS	M	54	69	6.8
Control #1	metastatic ovarian cancer	F	68	58.5	6.2
Control #2	lung cancer	F	52	44	6.7
Control #3	myocardial infarction	M	61	53	6.0
Control #4	renal cancer	F	77	21	6.2
Control #5	carcinomatous meningitis	M	54	30	6.6
Control #6	aortic sclerosis; infection	F	89	41	6.0
Control #7	myocardial infarction	M	67	41	5.6
Control #8	cancer; unknown primary	M	80	48.25	5.1
Control #9	gastric & bronchial carcinoma	F	58	21	6.0
Control #10	myocardial infarction	F	55	95	6.0
Control #11	prostate cancer	M	65	26	7.0
Control #12	breast cancer	F	85	45	5.8
Control #13	cerebral infarction	M	83	48	6.9
Control #14	renal cancer	M	78	30.5	5.4
Control #15	peritonitis & septicaemia	M	79	24	5.1
Control #16	cardiac problems & prostate cancer	M	80	55	6.4
Control #17	chronic obstructive pulmonary disease	F	84	35	6.1
Control #18	cerebral infarction	M	81	18	5.1
Control #19	myocardial infarction & pneumonia	M	89	84	5.9
Control #20	asthma attack	F	88	39	6.4
Control #21	cancer; unknown primary	F	80	3	6.4
Control #22	syringomyelia	M	55	24	5.6
Control #23	natural causes[d]	F	83	74	6.6

[a]PMD - postmortem delay [b]RIN - RNA integrity number [c]ALS - all ALS cases in this study were apparently sporadic
[d]Natural causes - details unavailable (clinically bipolar affective disorder)

Additional file1: Table S2. MIQE Checklist

MIQE checklist for authors, reviewers and editors.
All essential information (E) must be submitted with the manuscript. Desirable information (D) should be submitted if possible.
If using primers obtained from RTPrimerDB, information on qPCR target, oligonucleotides, protocols and validation is available from that source.

ITEM TO CHECK	IMPORTANCE	Comments
**EXPERIMENTAL DESIGN**		
Definition of experimental and control groups	**E**	Materials & Methods; Table S1
Number within each group	**E**	34 ALS & 23 controls
Assay carried out by core lab or investigator's lab?	D	Investigator's lab
Acknowledgement of authors' contributions	D	
**SAMPLE**		
Description	**E**	Frozen postmortem brain tissue
Volume/mass of sample processed	D	60 mg
Microdissection or macrodissection	**E**	macro
Processing procedure	**E**	Materials & Methods
If frozen - how and how quickly?	**E**	Frozen at -80 ^{o}C after post-mortem delay as detailed in Table S1
If fixed - with what, how quickly?	**E**	unfixed
Sample storage conditions and duration (especially for FFPE samples)	**E**	Stored at -80 ^{o}C in MRC brain bank
**NUCLEIC ACID EXTRACTION**		
Procedure and/or instrumentation	**E**	
Name of kit and details of any modifications	**E**	RNeasy Lipid Tissue kit (Qiagen)
Source of additional reagents used	D	Materials & Methods
Details of DNase or RNAse treatment	**E**	on-column DNase treatment (RNase-free DNase Set, Qiagen
Contamination assessment (DNA or RNA)	**E**	DNA contamination assessment by qPCR without RT step
Nucleic acid quantification	**E**	RNA quantification performed
Instrument and method	**E**	Qubit™ RNA BR assay (Thermo Fisher Scientific Inc)
Purity (A260/A280)	D	Assessed by NanoDrop™ (Thermo Fisher Scientific Inc)
Yield	D	
RNA integrity method/instrument	**E**	Agilent RNA 6000 Nano assay (Agilent Technologies, Inc.)
RIN/RQI or Cq of 3' and 5' transcripts	**E**	RIN values detailed in Table S1
Electrophoresis traces	D	Agilent RNA 6000 Nano assay (Agilent Technologies, Inc.)
Inhibition testing (Cq dilutions, spike or other)	**E**	ND†
**REVERSE TRANSCRIPTION**		
Complete reaction conditions	**E**	Materials & Methods
Amount of RNA and reaction volume	**E**	1 µg of RNA in a 20 µl reaction
Priming oligonucleotide (if using GSP) and concentration	**E**	2X RT Reaction Mix includes oligo(dT)$_{20}$ (2.5 µM) & random hexamers (2.5 ng/µL)
Reverse transcriptase and concentration	**E**	Invitrogen SuperScript III First-Strand

		Synthesis Supermix for qRT-PCR
Temperature and time	**E**	According to manufacturer's instructions
Manufacturer of reagents and catalogue numbers	D	SuperScript III First-Strand Synthesis Supermix for qRT-PCR<br>Thermo Fisher Cat No.11752-050
Cqs with and without RT	D*	See Results for details
Storage conditions of cDNA	D	cDNA stored at -20 ^{o}C
**qPCR TARGET INFORMATION**		
If multiplex, efficiency and LOD of each assay.	**E**	Not multiplex. PCR efficiencies given in Results
Sequence accession number	**E**	NA‡
Location of amplicon	D	Table 1
Amplicon length	**E**	Confirmed by electrophoresis & sequencing. Available on request
In silico specificity screen (BLAST, etc)	**E**	Yes
Pseudogenes, retropseudogenes or other homologs?	D	
Sequence alignment	D	
Secondary structure analysis of amplicon	D	
Location of each primer by exon or intron (if applicable)	**E**	NA‡
What splice variants are targeted?	**E**	NA‡
**qPCR OLIGONUCLEOTIDES**		
Primer sequences	**E**	Table 1
RTPrimerDB Identification Number	D	
Probe sequences	D**	NA‡
Location and identity of any modifications	**E**	none
Manufacturer of oligonucleotides	D	Eurofins Genomics & Qiagen
Purification method	D	
**qPCR PROTOCOL**		
Complete reaction conditions	**E**	Materials and Methods
Reaction volume and amount of cDNA/DNA	**E**	20 µl reaction with 2 µL cDNA
Primer, (probe), Mg++ and dNTP concentrations	**E**	0.25 µM each primer
Polymerase identity and concentration	**E**	Fast SYBR Green Master Mix (Thermo Fisher)
Buffer/kit identity and manufacturer	**E**	Fast SYBR Green Master Mix (Thermo Fisher)
Exact chemical constitution of the buffer	D	Fast SYBR Green Master Mix (Thermo Fisher)
Additives (SYBR Green I, DMSO, etc.)	**E**	Fast SYBR Green Master Mix (Thermo Fisher)
Manufacturer of plates/tubes and catalog number	D	Applied Biosystems MicroAmp Optical 96-well reaction plate. no. N8010560
Complete thermocycling parameters	**E**	Materials and Methods
Reaction setup (manual/robotic)	D	manual
Manufacturer of qPCR instrument	**E**	Applied Biosystems QuantStudio™ 5 thermocycler
**qPCR VALIDATION**		
Evidence of optimisation (from gradients)	D	

Specificity (gel, sequence, melt, or digest)	**E**	Confirmed by gel electrophoresis and sequencing
For SYBR Green I, Cq of the NTC	**E**	NTC Cq >45
Standard curves with slope and y-intercept	**E**	Available on request
PCR efficiency calculated from slope	**E**	Yes, see Results
Confidence interval for PCR efficiency or standard error	D	
r2 of standard curve	**E**	approximately 0.99
Linear dynamic range	**E**	NA
Cq variation at lower limit	**E**	ND
Confidence intervals throughout range	D	
Evidence for limit of detection	**E**	NA
If multiplex, efficiency and LOD of each assay.	**E**	Not multiplex
**DATA ANALYSIS**		
qPCR analysis program (source, version)	**E**	QuantStudio Design and Analysis software v1.4.3
Cq method determination	**E**	Automatic baseline setting and manual threshold setting
Outlier identification and disposition	**E**	NA
Results of NTCs	**E**	>45 cycles
Justification of number and choice of reference genes	**E**	Two reference gene transcripts used. Validation by qBase+ and RefFinder ranking of the nine candidates
Description of normalisation method	**E**	$2^{-\Delta\Delta Ct}$ method by normalisation against both *GAPDH* and *XPNPEP1*
Number and concordance of biological replicates	D	
Number and stage (RT or qPCR) of technical replicates	**E**	Materials and Methods
Repeatability (intra-assay variation)	**E**	Standard deviation of duplicates <0.2
Reproducibility (inter-assay variation, %CV)	D	
Power analysis	D	
Statistical methods for result significance	**E**	Materials and Methods

* Assessing the absence of DNA using a no RT assay is essential when first extracting RNA. Once the sample has been validated as DNA-free, inclusion of a no-RT control is desirable, but no longer essential.

‡NA = not applicable

** Disclosure of the probe sequence is highly desirable and strongly encouraged.

† ND = not done

Additional file1: Table S3. RefFinder rankings of candidate reference genes

	Ranking Order (Better--Good--Average)								
**Algorithm**	**1**	**2**	**3**	**4**	**5**	**6**	**7**	**8**	**9**
**Delta CT**	***XPNPEP1***	***CYC1***	***GAPDH***	***ACTB***	***SDHA***	***UBC***	***RPL13A***	***EIF4A2***	***YWHAZ***
**BestKeeper**	***UBC***	***SDHA***	***RPL13A***	***GAPDH***	***XPNPEP1***	***CYC1***	***ACTB***	***YWHAZ***	***EIF4A2***
**NormFinder**	***XPNPEP1***	***CYC1***	***GAPDH***	***ACTB***	***SDHA***	***UBC***	***RPL13A***	***EIF4A2***	***YWHAZ***
**GeNorm**	***SDHA | GAPDH***		***XPNPEP1***	***CYC1***	***ACTB***	***UBC***	***RPL13A***	***EIF4A2***	***YWHAZ***
**Recommended comprehensive ranking[a]**	***XPNPEP1***	***GAPDH***	***SDHA***	***CYC1***	***UBC***	***ACTB***	***RPL13A***	***EIF4A2***	***YWHAZ***

[a]The recommended comprehensive ranking generated by RefFinder is worked out based on the geometric mean of the stability ranking from all 4 algorithms; the comprehensive ranking lists *XPNPEP1* and *GAPDH* as the 2 most stable reference genes.
